# Managing Bone Infections Beyond Systemic Antibiotics: A Scoping Review

**DOI:** 10.3390/pathogens15020201

**Published:** 2026-02-11

**Authors:** Eleni Polyzou, Maria Gavatha, Dimitrios Efthymiou, Despoina Papageorgiou, Evangelia Ntalaki, Nikolaos A. Stavropoulos, Karolina Akinosoglou

**Affiliations:** 1 Department of Internal Medicine, University General Hospital of Patras, 26504 Rio, Greece; polyzou.el@gmail.com (E.P.); efthdim2@gmail.com (D.E.); dspn.pap96@gmail.com (D.P.); 2 Faculty of Medicine, University of Patras, 26504 Rio, Greece; gavatha.maria@yahoo.com; 3 Department of Obstetrics and Gynecology, University General Hospital of Patras, 26504 Rio, Greece; evangelia.ntalaki@gmail.com; 4 Second Department of Orthopaedic Surgery, School of Medicine, National and Kapodistrian University of Athens, Konstantopouleio General Hospital, 14233 Athens, Greece; nikstavropoulos@gmail.com; 5 Division of Infectious Diseases, University General Hospital of Patras, 26504 Rio, Greece

**Keywords:** osteomyelitis, bone and joint infections, local antibiotic delivery, biofilm-associated infection, immunomodulatory therapies

## Abstract

Bone infections, including osteomyelitis, prosthetic joint infections, and fracture-related infections, represent a persistent and growing clinical problem associated with substantial morbidity, mortality, and healthcare costs. Their management is complicated by limited bone vascularization, biofilm formation, intracellular bacterial persistence, dysregulated host immune responses and reduced antibiotic delivery to the infection site, which promote chronic infection and recurrence. The limitations of conventional treatment strategies based on surgical debridement and prolonged systemic antibiotic therapy, together with their association with antimicrobial resistance and systemic toxicity, have led to growing interest in alternative and adjunctive therapeutic approaches. Local antibiotic delivery systems, such as polymethyl methacrylate, calcium sulfate, hydroxyapatite-based composites, hydrogels, antibiotic-impregnated bone grafts, and nanoparticle carriers, enable high local antimicrobial concentrations while minimizing systemic exposure. From a different therapeutic perspective, immunomodulatory strategies, including mesenchymal stem cell-based therapies, cytokine-targeted interventions, bacteriophages, quorum-sensing inhibitors, and non-antibiotic antimicrobials, represent emerging approaches aimed at improving infection control and supporting bone regeneration. Advances in biomarker profiling, molecular diagnostics, and artificial intelligence-assisted analyses further support personalized approaches to diagnosis, monitoring, and treatment. Despite encouraging early results, clinical translation remains limited by methodological and regulatory challenges, underscoring the need for integrated, innovative treatment strategies.

## 1. Introduction

Bone infections are a general term that includes osteomyelitis, prosthetic joint infections (PJIs), and fracture-related infections (FRIs). Osteomyelitis, which can be classified as acute or chronic, is an infection involving the bone and surrounding structures caused by pyogenic microorganisms introduced via the bloodstream, fractures, or surgical procedures [1], whereas PJI, also known as periprosthetic infection, refers to an infectious process affecting the implanted joint prosthesis and the surrounding tissues [2]. Timing-based definitions of PJI vary across guidelines. Early postoperative infection is often described as occurring within 3–6 weeks after surgery, while the 2021 International Consensus Meeting on Musculoskeletal Infection considers infections within three weeks—and in some interpretations up to 90 days—as acute. These categories are not absolute, as hematogenous infections can present abruptly even long after surgery [3,4]. FRIs are infections that occur following a trauma incident, either as a consequence of the initial injury, open fracture, or during the course of fracture treatment [5]. There is currently no universally accepted or precise definition for FRI, as reported by recent surveys and reviews showing inconsistency in its terminology and diagnostic criteria [6]. In an effort to establish clearer diagnostic guidance, the international FRI consensus group proposed standardized diagnostic criteria, distinguishing between confirmatory signs, such as purulent drainage, sinus tract, or ≥2 positive deep tissue cultures and suggestive clinical, laboratory, or radiological findings [7]. In clinical practice, FRIs are often categorized based on the time from fracture or surgical intervention to infection onset, being classified as early (<2 weeks), delayed (2–10 weeks), or late (>10 weeks) infections [8]. 

The incidence of osteomyelitis in the United States was reported at 21.8 cases per 100,000 person-years, with higher rates observed in men and older adults. Similar reports from Europe indicate an incidence of approximately 15–17 cases per 100,000 inhabitants, with a 10.4% increase observed between 2008 and 2018 [9]. The majority of cases were chronic, predominantly affecting men and older adults, with the lower extremities being the most common site of infection [9]. Bone infections are associated with substantial morbidity, mortality, and economic burden. Evidence shows that chronic osteomyelitis significantly increases long-term mortality risk in elderly patients, especially in men and those with multiple comorbidities, with the risk persisting for years after diagnosis [10]. Similarly, large-scale epidemiological data from France demonstrate that bone and joint infections (BJIs) require repeated and prolonged hospitalizations, contribute to high morbidity, and impose considerable healthcare costs, exceeding €250 million annually [11]. These findings highlight that, bone infections remain a significant and growing clinical concern, particularly in aging populations. 

Standard management of osteomyelitis usually involves surgical debridement followed by prolonged administration of systemic antibiotics at high doses. Nevertheless, this strategy often proves inadequate and may contribute to the emergence of antibiotic-resistant bacteria [12,13]. Furthermore, their use at the concentrations required for eradication can lead to systemic toxicity [13,14]. Several limitations of the conventional use of antibiotics include the lack of robust clinical evidence, as few randomized controlled trials have compared the efficacy of different agents in treating bone and joint infections [13]. The variable ability of antibiotics to penetrate bone tissue and the lack of the ability to effectively target bacteria that evade immune detection and persist within osteocytes further complicates treatment [13,14]. Additionally, while achieving minimum serum bactericidal concentrations has been explored, it is no longer routinely recommended due to uncertain clinical relevance [13]. The growing prevalence of antibiotic-resistant organisms, including methicillin-resistant *Staphylococcus aureus* (MRSA) and vancomycin-resistant enterococci (VRE), significantly reduces the effectiveness of standard therapies. Some infections require combination regimens, which increase the potential for adverse drug interactions and side effects. The need for prolonged antibiotic courses and frequent surgical intervention also adds to treatment complexity, increasing the risk of toxicity, adverse effects, and poor patient compliance [13,14].

Considering all these factors, novel non-systemic and non-surgical strategies are becoming increasingly important in addressing the persistent challenges of bone infection management. While previous reviews have largely focused on systemic antimicrobial therapies or have examined individual local antibiotic or antimicrobial approaches in isolation, the present review adopts a broader and integrative perspective by exploring the full spectrum of available non-systemic and non-surgical strategies for the management of bone infections. The literature discussed includes experimental studies, clinical investigations, and existing reviews, which are synthesized within a conceptual analytical framework that considers the underlying mechanisms of action, modes of delivery, and degree of translational development. Through this structured approach, the present review aims to clarify the current state of the field, identify knowledge gaps and unresolved controversies, and highlight emerging strategies with potential relevance for future therapeutic applications (methodological details are provided in the Supplementary File S1). 

## 2. Causes of Difficulty in Treating Osteomyelitis

### 2.1. Pathogen Factors

Pathogens can reach bone tissue and adhere to soft tissues, bone or implants through binding to extracellular matrix (ECM) proteins via recognition of adhesive matrix molecules, such as collagen-binding proteins and bone sialoproteins. To survive, multiply and protect themselves from invasion and clearance by immune cells, pathogens use different mechanisms leading to chronicity of bone infections (latent or recurrent) and to treatment failure. For example, *S. aureus* can form microcolonies known as staphylococcal abscess communities, a structure surrounded by fibrin network, in order to hide from host immune system. Biofilm formation is another way for pathogens to avoid the immune response and antibiotics by living in reduced levels of oxygen and maintaining low metabolism ratio [15,16]. In order to form a friendly microenvironment to multiply, pathogens form an extracellular polymeric substance matrix composed of self-produced polysaccharides and proteins and possibly extracellular DNA from dead bacterial cells [15].

Except for developing small communities and extracellular boundaries, pathogens can also invade the existing osteocyte lacuna-canalicular networks in the submicron channels buried deep within the dense mineral matrix of cortical bone, so that they can hide from host immune response and lead to chronic inflammation [17,18]. Bentley et al. have shown that, *S. aureus* can reduce its diameter to 20% of its native size and shift from a rounded to a rod shape, orienting perpendicularly to enter the canaliculus and anchor and propel the developing daughter cell into submicron bone channels [17].

Pathogens can also utilize a wide range of toxins including exfoliative toxins, pore-forming toxins, and superantigens, that target host cells. For example, the superantigen toxic shock syndrome toxin-1 promotes osteoclastogenesis and bone resorption, while pore-forming toxins such as leukotoxins (e.g., Panton–Valentine leukocidin), α- and β-hemolysins, and phenol-soluble modulins disrupt the membrane integrity of osteoclasts, osteoblasts, and PMNs [19].

Extracellular pathogens can also invade and persist intracellularly in fibroblasts, osteoblasts, and phagocytes by expressing fibronectin-binding proteins (A and B) that bind fibronectin and interact with α5β1 integrins on macrophages or neutrophils. For intracellular survival, pathogens may enter a dormant state, such as the *S. aureus* small-colony variant phenotype, characterized by electron transport mutations, slow growth, reduced metabolism, and functional resistance [15,20,21].

Pathogen–bone interactions disrupt bone homeostasis, with osteolysis predominating over bone formation. Consequently, bone resorption, cortical thickening, and periosteal new bone apposition are key imaging findings in osteomyelitis. Periosteal bone formation typically appears early as a response to inflammation, whereas cortical osteolysis results from bacterial spread through Haversian and Volkmann canals. In chronic disease, cytokine release drives osteoclastic resorption, fibrous tissue ingrowth, and peripheral reactive bone deposition, forming an involucrum around devitalized infected bone [22,23,24,25,26]. Bone resorption occurs through indirect immune-mediated effects and direct bacterial damage [16]. Infection-induced immune mediators disrupt osteoblast mineral deposition and enhance osteoclast activity, with oxidative stress driven by TNF-α and IL-1β further impairing bone integrity [27]. *S. aureus* promotes osteoclastogenesis via staphylococcal protein A-mediated activation of TNF-α and EGFR signaling, inducing RANK-L expression and suppressing osteoblast differentiation markers [15,27,28,29]. RANK-L upregulation also occurs following bacterial internalization by osteoblasts and TLR4 activation in PMNs [15,27,28,29]. Additionally, small colony variants induce osteoblast apoptosis through the TRAIL/caspase pathway [20].

### 2.2. Blood Flow Impairment

Bone tissue is a highly metabolic tissue demanding abundant vascular supply, estimated to use approximately 10–20% of resting cardiac output [30]. However, slow flow of blood, particularly seen at the vascular loops in areas like metaphysis near the epiphyseal plates or the vertebral bodies, plays a crucial role for hematogenous osteomyelitis deposition of microbes. When combined with the immunocompromising state and vasculopathy caused by diabetes mellitus, the deposition becomes even easier [20]. This vascular impairment is crucial in cases of diabetic foot ulcer complicated with diabetic foot infection (DFI) and osteomyelitis, where mechanisms of repetitive trauma and loss of sensitivity due to peripheral neuropathy are also present [31]. Microorganisms affect the periosteum and even form subperiosteal abscesses, impairing the blood supply while simultaneously spread within the bone causing segmental bone necrosis, areas known as bone sequestrum [26]. This impaired vascularity in regions of infected bone, irrespectively of the presence of diabetes, either due to complete loss of vasculature or due to relative hypoperfusion, is a critical factor for antibiotic treatment failure. Not only devitalized areas of infected bone (bone sequestra), but also surrounding areas of living bone compromised by the relative hypoperfusion are deprived from leucocytes and sufficient antibiotic concentrations. This leads to a hostile environment with low pH, excessive cellular damage and degrading proteases. Moreover, hypoxic conditions help the growth of anaerobic organisms, inhibit collagen synthesis and osteogenesis and reduce the ability of neutrophils to generate reactive oxygen species to kill bacteria. Traditionally, surgical debridement of necrotic tissue and restoration of normal vascularity to the point of healthy bleeding (paprika sign) was the main therapeutic choice with less invasive options like hyperbaric oxygen therapy (HBOT) or sericin nanoparticles as vascular endothelial growth factor (VEGF) nanocarriers being a suitable option too [32,33].

### 2.3. Biofilm Formation

A major challenge in bone infections is bacterial biofilm formation, particularly on devitalized bone such as sequestra, and on implants where it can lead to periprosthetic joint infection and often necessitate prosthesis removal [20,32]. Biofilms are structured, sessile microbial communities irreversibly attached to surfaces and embedded in a self-produced extracellular polymeric matrix composed of polysaccharides, proteins, extracellular DNA, and water [34,35]. This matrix enables mixed-species bacteria to evade host immune defenses, coordinate behavior through quorum sensing, and survive hostile environments [20,32,36]. Within biofilms, metabolic and oxygen gradients generate slow-growing or dormant persister cells with reduced antibiotic susceptibility [35]. Biofilm development progresses from surface attachment to maturation into a three-dimensional structure with biofilm-specific gene expression and enhanced virulence, followed by dispersal that sustains chronic infection [34]. Antibiotic recalcitrance arises from limited antimicrobial penetration through the extracellular matrix, reduced bacterial metabolic activity, and increased horizontal gene transfer within the biofilm microenvironment, resulting in resistance levels far exceeding those of planktonic bacteria [34,37,38].

**Figure 1:**
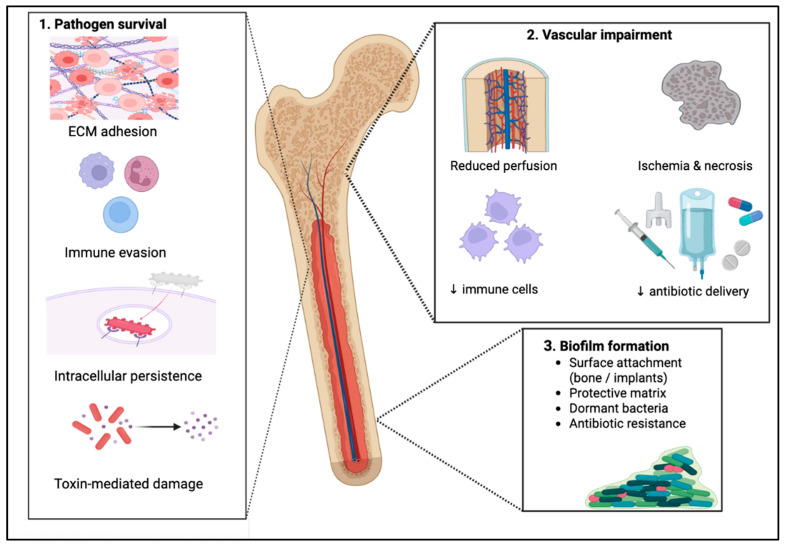
Figure illustrating the different ways bone infections lead to chronicity and persistency. Pathogens adhere to bone and implant surfaces through binding to extracellular matrix (ECM) (**1**). Bone regions with compromised vascularization (sequestra) create a favorable niche for microbial colonization by limiting immune surveillance and antibiotic penetration. Pathogen persistence is further supported by immune evasion mechanisms, including toxin production that damages host cell integrity and the ability to invade and survive intracellularly within fibroblasts, osteoblasts, and phagocytic cells (**2**). Finally, a major resistance factor is the formation of sessile microbial communities, also known as biofilm, that are attached to surfaces and embedded in a polymeric matrix favoring low metabolic rate and antibiotic resistance (**3**).

Figure 1 illustrates the various mechanisms through which bone infections develop chronicity and persist, including biofilm formation, vascular impairment, and intracellular bacterial survival.

## 3. Non-Systemic Antibiotic Therapy

Considering these different defensive mechanisms in the setting of biofilms and bone infections, antibiotic therapy is a challenging and concerning area not only for the cure of these infections but also for the rise of antimicrobial resistance generally. The structure of biofilm’s microenvironment with the presence of exopolysaccharides (EPS) resistance mechanisms, the slow growth rate within the biofilm, the horizontal gene transfer, the presence of small-colony variants (SCVs) that can escape from antibiotics and hosts immune response, the impaired vascularity and bone sequestra, which makes the antibiotic penetration to the infection site even more problematic, are a few reasons why antibiotic therapy alone seems a less viable solution for the therapy of bone infections raising questions about other alternative or adjunctive strategies to the traditional antibiotic therapy and necessitating the creation of new cutting-edge techniques.

### 3.1. Local Antibiotic Delivery Systems

Local antibiotic delivery systems have emerged as an effective strategy in orthopedic trauma care, providing high local antibiotic concentrations at the infection site while minimizing systemic toxicity, and offering a promising alternative to conventional systemic therapy for the prevention and treatment of fracture-related infections [39]. The available local antibiotic delivery systems include polymethyl methacrylate (PMMA), calcium sulfate (CaS), hydroxyapatite (HA), hydrogels, antibiotic-impregnated bone grafts (AIBGs), and nanoparticles. Table 1 provides a summary of the key characteristics and features of the various local antibiotic delivery systems.

#### 3.1.1. Polymethyl Methacrylate (PMMA)

PMMA is a non-resorbable bone cement used in orthopedic practice [40]. It is originally made from methyl methacrylate (MMA) monomers, which bind through radical polymerization to form PMMA [41]. It can be loaded with antibiotics to help reduce bone infections [40]. In terms of its material abilities, PMMA shows good biocompatibility with human tissues but possesses lower mechanical strength than natural bone, including reduced resistance to compression, fatigue, and tension [42]. PMMA bone cements are classified into low-, medium-, and high-viscosity formulations, each with distinct handling properties [43]. Low-viscosity cements have a prolonged mixing phase but a short working phase, requiring strict adherence to timing, whereas high-viscosity cements set more rapidly yet provide a longer working window for application. Optimal viscosity must balance adequate penetration into bone, while preventing undesired mixing with blood or debris [43]. Although some studies have shown that medium- and high-viscosity PMMA cements demonstrate comparable antibiotic elution profiles, suggesting that viscosity alone may not substantially alter antimicrobial performance, further research is still required to clarify their *in vivo* behavior and clinical impact [44].

When it comes to antibiotic options, several parameters should be taken into account, as not all antibiotics are suitable for incorporation into PMMA [45]. The antibiotics must be heat-stable to withstand the exothermic polymerization reaction, water-soluble to allow elution from the cement, and available in powder form for proper mixing [42,45]. Moreover, they should possess broad-spectrum (including Gram-positive and Gram-negative bacteria), bactericidal activity with a low risk of resistance or allergic reactions [42,45]. The antibiotics commonly used in PMMA bone cement include gentamicin, tobramycin, and vancomycin, which provide broad-spectrum antibacterial coverage and help reduce the risk of resistance development [45,46]. Additionally, other antibiotics such as moxifloxacin, ciprofloxacin, tetracycline, clindamycin, daptomycin, ertapenem, meropenem, and cefotaxime can also be incorporated into PMMA [42,47,48]. Beyond antibacterial therapy, antifungal agents like amphotericin B may be added to PMMA formulations to manage fungal prosthetic joint infections [49]. Combining antibiotics within PMMA, most commonly vancomycin with gentamicin or tobramycin, broadens antimicrobial coverage and may enhance efficacy through synergistic effects [45]. Alternative combinations, including the addition of agents such as daptomycin, allow treatment of resistant organisms like VRE [50].

Data from cohort studies reveal that antibiotics containing PMMA beads are effective in the treatment of chronic osteomyelitis and in preventing the recurrence of infection [51]. These beads allow for prolonged local antibiotic therapy, maintaining high drug concentrations directly at the infection site [52]. However, outcomes may vary depending on factors such as bacterial resistance, local tissue conditions, and the choice of antibiotic combination [51]. When combined, vancomycin-loaded calcium sulfate (CaS) and vancomycin-loaded PMMA showed synergistic effects, achieving more effective control of infection in the treatment of chronic osteomyelitis [53]. This combination provided both immediate structural stabilization from the non-biodegradable PMMA and a higher local antibiotic concentration from the biodegradable CaS, resulting in improved infection eradication rates and reduced need for reoperation compared with PMMA alone [53]. Interestingly, from a different perspective, antibiotic-loaded bone cement (ALBC) appeared to exert notable immunomodulatory properties following total knee arthroplasty [54]. Patients treated with ALBC demonstrated significantly lower serum interleukin-6 (IL-6) levels at 72 h postoperatively compared to those who received standard bone cement, suggesting that ALBC may help to moderate the early postoperative inflammatory response [54]. Although joint fluid IL-6 levels and functional outcomes such as Knee Society Scores did not differ significantly between groups, the observed reduction in systemic inflammatory markers indicates that, ALBC may offer benefits beyond infection prevention, potentially contributing to improved immunological balance and recovery following surgery [54].

Major limitations of antibiotic-loaded PMMA include variable antibiotic elution influenced by mechanical and drug-specific factors, along with restricted antibiotic selection, as liquid formulations impair polymerization and weaken cement strength [55]. Variability in mixing techniques and excessive antibiotic loading (>10%) can compromise mechanical stability, increasing the risk of spacer-related complications such as breakage, dislocation, or neurovascular compression [56,57]. Manual mixing may also cause material abrasion and potential contamination during preparation [58]. Additionally, PMMA spacers typically require later removal and replacement with bone grafts when used for bone defects [59]. Of particular concern is the emergence of antimicrobial resistance due to subinhibitory antibiotic concentrations during prolonged implantation, leading to recolonization or resistant pathogens, including gentamicin-resistant coagulase-negative staphylococci [60,61,62,63].

#### 3.1.2. Calcium Sulfate (CaS) 

CaS is a biocompatible and biodegradable material widely used in orthopedics and biomedical engineering, valued for its role in bone regeneration and its ability to function as a local antibiotic carrier [64]. Due to its gradual dissolution, CaS can achieve sustained release of high local antibiotic concentrations at the site of infection, typically over 3–6 weeks in soft tissue and 6–12 weeks in bone, —allowing effective treatment of biofilm-associated infections without systemic toxicity [65,66]. Beyond vancomycin and tobramycin, CaS can deliver a broad range of antibiotics, including amikacin, meropenem, fosfomycin, minocycline, cefazolin, and dalbavancin [67].

Clinical studies suggest improved infection-free outcomes in patients with favorable host and limb conditions, though effectiveness is reduced in immunocompromised individuals or those with poor wound healing [68]. CaS is cost-effective and has demonstrated efficacy in reducing postoperative infections, including in diabetic patients, diabetic foot osteomyelitis, and chronic knee PJI treated with two-stage revision [69,70,71,72]. When combined with PMMA, CaS enhances antibiotic release and antibacterial activity compared with PMMA alone [73]. While large cohort studies show limited benefit in early or chronic PJI, evidence suggests reduced reoperation rates and improved infection control in selected cases, particularly acute hematogenous infections [74].

Reported complications include hypercalcemia, rare but potentially serious due to rapid material degradation, as well as, delayed wound healing [75,76,77]. The latter is attributed to the rapid degradation of CaS that not only elevates calcium ion concentrations, but also potentially delays wound healing due to the resulting imbalance in the local microenvironment [78]. Low compressive strength limits its use in load-bearing defects [78], and postoperative wound drainage remains the most common adverse effect, influenced by bead location and patient- and procedure-related factors, though it occurs less frequently with synthetic formulations [79,80,81].

#### 3.1.3. Hydroxyapatite (HA) 

HA is the inorganic phase of bone, a calcium phosphate compound that closely resembles the mineral component of natural bone tissue [82]. It is a biocompatible and bioactive material widely used in medical applications such as bone grafts, reconstruction, and regeneration. Its ability to absorb water and interact with biological fluids makes it an excellent carrier for water-soluble therapeutic agents in the treatment of bone pathologies [82]. 

Ιn bone infections, HA is commonly combined with CaS to form biphasic CaS/HA composites that enhance antibacterial activity, support bone regeneration, and function as absorbable bone void fillers [83]. Antibiotic-loaded CaS/HA combinations have demonstrated effective eradication bone including osteomyelitis in diabetic patients with commercial formulations such as Cerament^®^ showing favorable limb-salvage outcomes in diabetic foot osteomyelitis [83,84,85,86]. Notably, gentamicin-loaded CaS/HA can achieve high local antibiotic concentrations effective even against pathogens resistant to systemic therapy [87].

Vancomycin and gentamicin are the most extensively studied antibiotics in HA-based delivery systems, while combinations including tobramycin have shown prolonged antibacterial activity, with sustained release for up to 35 days and strong efficacy against *S. aureus* [86,88]. Other agents such as cephalexin and norfloxacin also demonstrate sustained release *in vitro* [89].

However, HA alone tends to crystallize and coagulate rapidly *in vivo*, limiting its performance and prompting combination strategies to improve stability [90]. Despite promising results, antibiotic-loaded CaS/HA composites may still be associated with wound complications, persistent infection, and the need for revision surgery in selected cases [91].

#### 3.1.4. Hydrogels

Hydrogels can be categorized according to several complementary criteria, including their source of origin, cross-linking mechanism, ionic charge, method of preparation, and responsiveness to external stimuli. Based on their source, hydrogels are commonly classified as natural, synthetic, or hybrid systems. Natural hydrogels are derived from biological materials such as proteins and polysaccharides, synthetic hydrogels are produced through chemical polymerization of defined monomers and crosslinking agents, and hybrid hydrogels combine natural and synthetic components to optimize physicochemical and biological properties [92,93]. Hydrogels exhibit high biocompatibility due to their structural resemblance to the human ECM and their elevated water content [94]. Their low toxicity, biodegradability, viscoelastic nature, and ability to conform to diverse anatomical surfaces make them highly suitable and effective as localized drug delivery systems [95]. Of most importance is the hydrogel’s ability to guide the formation of new bone tissue by supporting cell attachment, proliferation, and differentiation [96]. In addition, the porous structure of hydrogels and their compatibility with aqueous biological environments allow efficient drug incorporation and controlled release, further supporting their use as biocompatible delivery systems [97]. Moreover, hydrogels can be formulated in different physical forms, such as films, slabs, microparticles, and nanoparticles, which broadens their applicability in biomedical applications [97]. Hydrogels can be classified into those with inherent antimicrobial activity and those loaded with antimicrobial agents, depending on their composition [98]. Inherently antimicrobial hydrogels include peptide-based, amphoteric ion, and polysaccharide-based systems, where the polymer itself confers antibacterial activity [98]. In contrast, drug-loaded hydrogels incorporate antibiotics, biological extracts, metal nanoparticles, or antimicrobial peptides to enhance efficacy and enable controlled local release [98]. From a pharmacokinetic aspect, antibiotic-loaded hydrogels provide targeted, sustained drug delivery that maintains therapeutic concentrations at the infection site while minimizing systemic exposure, improving efficacy, and potentially reducing bacterial resistance, with additional applications in wound care and tissue engineering [99].

Commonly incorporated antibiotics include ciprofloxacin, gentamicin, vancomycin, nitroimidazoles, and sulfonamides, with multiple formulations demonstrating strong activity against *S. aureus* (including MRSA), *P. aeruginosa*, and *E. coli* [100,101,102,103,104,105,106,107]. 

Overall, natural hydrogels offer superior biological activity and favorable interactions with bone-related cells but are often limited by insufficient mechanical stability, whereas synthetic hydrogels provide enhanced structural integrity and durability at the expense of intrinsic bioactivity, highlighting the need for composite systems that integrate the advantages of both approaches [108]. Several material-specific limitations of hydrogel-based drug delivery systems have been reported in the literature, including challenges related to mechanical performance, degradation control, fabrication requirements, and safety considerations, depending on hydrogel composition and formulation [108].

Clinically, antibiotic-loaded hydrogels used as fast-resorbable implant coatings significantly reduce surgical site and periprosthetic joint infections without impairing osseointegration or causing adverse effects, and show promising results in fracture fixation and revision procedures [109,110,111,112,113,114,115]. However, despite encouraging findings, there remains a lack of robust clinical evidence supporting their therapeutic efficacy [116].

#### 3.1.5. Antibiotic-Impregnated Bone Grafts (AIBGs)

Antibiotic-impregnated bone grafts (AIBGs) represent an advanced approach in bone reconstruction, combining the structural and regenerative benefits of bone grafts with localized antibiotic delivery to effectively prevent and treat infection at the graft site [117]. Cancellous bone is typically preferred due to its osteoconductive properties and porous structure that are associated with a lower risk of necrosis and infection persistence than the cortical bone [118].

A major advantage of AIBGs is the ability to incorporate a wide range of antibiotics, enabling targeted therapy based on microbial susceptibility; however, combining multiple agents may alter elution dynamics and antibacterial efficacy [118,119].

Clinical studies report high infection eradication rates and successful graft incorporation, allowing simultaneous infection control and bone reconstruction with durable infection-free outcomes, particularly in revision arthroplasty and implant-related surgeries [120,121]. In trauma settings, AIBGs have demonstrated improved infection control and bone healing compared with conventional grafts, with sustained success over long-term follow-up [122]. Although impaction bone grafting in two-stage hip revisions may be prone to reinfection, evidence suggests comparable long-term outcomes with or without antibiotic loading [123]. Emerging techniques, such as iontophoresis, further enhance antibiotic delivery into allograft bone, achieving high local concentrations and effective reinfection prevention in revision arthroplasty [124,125].

#### 3.1.6. Nanoparticles

Nanoparticles are materials engineered at the atomic or molecular scale, typically ranging from 1 to 100 nanometers in size [126]. Their extremely small dimensions grant them unique structural, chemical, and biological properties that distinguish them from other materials [127,128]. Due to their nanoscale size, nanoparticles can move freely within the human body and interact efficiently with biological systems. These characteristics make them excellent drug delivery systems, as they can encapsulate or bind therapeutic agents, enabling targeted delivery to specific tissues, controlled drug release, and enhanced bioavailability while minimizing systemic side effects [129,130]. As biodegradable materials, they eliminate the need for secondary removal surgeries and reduce patient risk, while their nanoscale structure enables sustained antibiotic release and simultaneously supports bone regeneration at the infection site [90]. Nanomaterials are increasingly investigated for the management of bone infections, enabling advances in diagnostic strategies, antimicrobial sterilization, targeted drug delivery, and the treatment of chronic disease [131]. 

Nanoparticles used in biomedicine can be classified according to the materials employed in their synthesis, as material composition critically determines their physicochemical properties, biological interactions, and safety profiles [132]. Major nanoparticle platforms include metallic nanoparticles (e.g., gold, silver, iron oxide), lipid-based systems such as liposomes and polymeric nanoparticles, natural or synthetic, polymer-based nanoparticles particularly those based on biodegradable polymers such as PLGA [132,133]. Metallic nanoparticles combat bone infections via membrane disruption, metal ion release, and ROS generation, leading to broad-spectrum antibacterial and antibiofilm activity while supporting bone repair processes [134], whereas lipid nanoparticles are biocompatible and biodegradable nanocarriers composed of lipid matrices stabilized by surfactants, widely used for the delivery of therapeutic and diagnostic agents, including small molecules and nucleic acids, due to their ability to encapsulate both hydrophobic and hydrophilic compounds and enable controlled and targeted drug delivery [135,136]. In bone infections, polymeric nanoparticles act as biodegradable local drug delivery systems that enable targeted administration of antibiotics, allowing sustained release with high penetrability into infected bone and surrounding soft tissue, while reducing drug load, systemic exposure, and toxicity [137]. Among synthetic nanoparticle platforms, ceramic nanoparticles composed of inorganic materials such as silica, titania, and alumina act as stable nanocarriers that protect entrapped drugs, proteins, or enzymes from denaturation by external pH and temperature due to their high mechanical strength and minimal swelling or porosity changes [138].

Incorporating antibiotic-loaded nanoparticles into PMMA bone cement has shown promise in reducing bacterial growth and preventing infection by improving local drug stability and release kinetics, enhancing antimicrobial efficacy, and reducing the risk of bacterial colonization associated with conventional PMMA systems [139]. Chitosan-based nanoparticles enhance antibacterial efficacy while preserving biocompatibility and mechanical stability [140] and ceramic–polymer hybrid nanoparticles loaded with antibiotics such as nafcillin or levofloxacin enable controlled release and effective disruption of *S. aureus* biofilms [141]. Nanoparticles have also been explored as alternative carriers to address limitations of HA, with HA–PLGA nanoparticle systems providing sustained antibiotic release, maintained antibacterial activity, and support for bone regeneration [142,143,144]. Silver nanoparticles exhibit broad-spectrum antimicrobial activity and effectively inhibit biofilm formation, making them particularly attractive for incorporation into orthopedic implants, bone cement, and surface coatings to reduce implant-associated infections caused by pathogens such as *Escherichia coli* (*E. coli*) and *S. aureus* [145,146]. Of interest, lipid–polymer hybrid nanoparticles loading linezolid showed ~60% encapsulation efficiency, controlled drug release, strong bone-targeting affinity when decorated with alendronate, prolonged local accumulation in bone with minimal distribution to remote organs, and significantly higher antibacterial efficacy against MRSA, including biofilm and intracellular forms, compared with free linezolid [147].

*In vivo* studies confirm these benefits, as PLGA-PEG nanoparticle matrices demonstrated effective antibacterial activity against *S. aureus* while promoting bone healing [148]. Clinically, antibiotic-loaded nanoparticles, including bioabsorbable tobramycin-impregnated calcium sulfate particles, achieved high infection eradication rates in posttraumatic osteomyelitis although complications such as refracture, recurrence of infection, persistent nonunion, superficial wound necrosis, and transient sterile draining sinuses were reported in such cases [149]. Other potential side effects of nanoparticles include systemic toxicity and cytotoxic responses arising from protein adsorption, interactions with blood components, cellular uptake by macrophages or fibroblasts, local tissue accumulation with pseudocapsule formation, and translocation to major organs such as the lung, liver, and spleen, often mediated by inflammatory responses and oxidative stress [150,151]. In terms of cost, the widespread clinical translation of nanoparticle-based systems remains limited by complex and imperfect large-scale manufacturing processes, high production expenses, and scale-up challenges [152,153].

### 3.2. Immunomodulatory Approaches

As bone infections are characterized by dysregulated immune responses, immunotherapy offers a novel approach to restoring immune balance and enhancing infection control. In osteomyelitis, excessive inflammation, immune checkpoint overexpression, and disrupted immune signaling impair host defense and promote bone loss through increased osteoclast activity [154]. Pathogens like *S. aureus* evade immune eradication by forming biofilms, invading host cells, and modulating immune responses, contributing to persistent and recurrent disease [155]. Pro-inflammatory interleukins, particularly IL-1, are central to disease pathogenesis, as polymorphisms in IL1RN and IL1B are associated with increased susceptibility to osteomyelitis and *S. aureus* infection, indicating that host inflammatory variability influences disease outcome [156]. Additionally, antibiotics can modulate local immune responses; agents such as cefuroxime more effectively suppress pro-inflammatory cytokines, including IL-1α and IL-6, which may enhance infection resolution and bone healing [157].

#### 3.2.1. Mesenchymal Stem Cells (MSCs)

MSCs are increasingly recognized as a therapeutic strategy in bone regeneration due to their capacity to differentiate into osteogenic lineages, modulate inflammation, and migrate to sites of injury [158]. By differentiating into osteoblasts, MSCs promote bone formation and mineralization, directly restoring skeletal integrity, whereas differentiation into chondrocytes supports cartilage scaffold formation during the early stages of fracture healing, providing structural stability and guiding subsequent bone regeneration [159]. Their response to mechanical stimuli by proliferating and differentiating into osteoblasts, is also essential for maintaining bone strength, density, and adaptation to physical demands [160]. In addition, MSCs secrete growth factors and cytokines that regulate inflammation, stimulate angiogenesis, and establish a pro-regenerative microenvironment [161]. 

MSCs exhibit strong immunomodulatory properties, regulating both innate and adaptive immunity. In response to inflammatory mediators such as IL-1, TNF-α, and IFN-γ, they secrete PGE-2, TGF-β1, IL-4, IL-6, IL-10, and IL-1Ra, suppressing immune cell activation and limiting inflammation. This results in reduced activity of macrophages, dendritic cells, NK cells, and lymphocytes, and promotes a shift from pro-inflammatory Th1 and M1 phenotypes toward anti-inflammatory Th2 and M2 states, facilitating tissue repair [162,163]. 

MSCs can be isolated from multiple tissues, including bone marrow, adipose tissue, skeletal muscle, umbilical cord, umbilical cord blood, placenta, Wharton’s jelly, and amniotic fluid [164]. Clinically, MSC-based therapies have been evaluated in, fracture healing, fracture nonunion, bone defects, and avascular necrosis [165]. Meta-analyses indicate that, MSC therapy significantly improves healing rates, shortens union time, and reduces complication compared with standard bone grafting, supporting its role as an adjunct treatment for nonunion fractures [166]. Preclinical studies consistently show enhanced bone regeneration, while clinical trials report improved outcomes with acceptable safety profiles [167]. Scaffold-based approaches combining MSCs with endothelial progenitor cells further promote angiogenesis and bone formation in critical-sized defects [168]. Autologous bone marrow MSC and culture-expanded osteoblasts have also demonstrated accelerated fracture union without serious adverse events [169,170].

Despite these advances, MSC therapies face limitations, including limited capacity to generate complex, organized tissues, inefficient homing to injury sites, and variability due to donor characteristics, cell source, and culture conditions. Lack of standardized protocols for MSC isolation, expansion, storage, and delivery further impairs reproducibility and clinical translation [171]. Ethical issues related to consent, cell sourcing, transparency, and equitable access also require ongoing regulatory oversight to ensure responsible clinical implementation [171,172].

#### 3.2.2. Interleukins and Macrophages

Interleukins play a critical role in bone infections by regulating the complex balance between inflammation, immune response, and bone remodeling; thus, targeting these cytokines may offer promising therapeutic strategies to control infection-induced inflammation and promote bone healing [173].

IL-6 antagonists may have a significant role in promoting bone healing, as supported by growing experimental and clinical evidence. Numerous studies have demonstrated that, inhibition of IL-6 signaling enhances bone regeneration by stimulating osteoblast activity, increasing bone formation rates, and promoting revascularization within necrotic bone tissue [174]. In parallel, transplantation of IL-10-overexpressing bone marrow-derived mesenchymal stem cells reduces inflammation, enhances osteogenesis, and improves mechanical properties of healing bone, particularly under diabetic conditions [175]. However, interleukin-targeted therapies may cause profound immunosuppression, increasing susceptibility to endogenous infections and related complications [176].

Monocytes also play a dual role in host defense and tissue regeneration, making them attractive therapeutic carriers in osteomyelitis. Their intrinsic capacity to home to infected tissue and differentiate into macrophages can be exploited to deliver targeted antibacterial and immunomodulatory therapies, thereby supporting infection control and bone healing [177].

#### 3.2.3. Vaccination

Immunization against antibiotic-resistant pathogens such as *S. aureus*, particularly MRSA, has been explored as an alternative strategy to reduce infection burden and dependence on antibiotics [178]. However, vaccine candidates targeting *S. aureus* have thus far failed to demonstrate effective clinical protection, with several trials showing limited efficacy or unfavorable outcomes [179]. In preclinical animal models, vaccine candidates based on α-toxin, capsular polysaccharides, ClfB, or multicomponent formulations conferred protection against sepsis, pneumonia, nasal colonization, and abscess formation, supporting the potential of multivalent, adjuvanted approaches to induce humoral and cellular immunity [180,181]. Conversely, phase II and phase III clinical studies —including capsular polysaccharide conjugate vaccines (serotypes 5 and 8) and the four-antigen SA4Ag formulation—elicited robust antibody responses but failed to provide sustained or meaningful protection against *S. aureus* infection in humans [182,183]. Furthermore, vaccination did not reduce nasal carriage and in some cases was associated with new *S. aureus* colonization in previously uncolonized individuals [184].

#### 3.2.4. Phages and Anti-Virulence Therapies

Bone infections constitute a significant cause of morbidity and mortality and are difficult to treat due to the limited vascularization of bone tissue, which restricts immune cell access and antibiotic penetration [185,186]. Beyond these anatomical constrains, bacterial pathogenicity is further regulated by Quorum Sensing (QS), a cell density-dependent communication system that coordinates gene expression through secreted autoinducers. In Gram-positive bacteria, QS controls key functions such as virulence regulation, motility, and host adaptation [187]. *S. aureus* QS network is mediated by the accessory gene regulator (Agr) system, which is activated by the accumulation of an autoinducing peptide (AIP). Upon reaching a threshold concentration, AIP binds the histidine kinase AgrC, triggering phosphorylation of the transcription factor AgrA. Activated AgrA induces expression of the agr operon and RNAIII, thereby upregulating multiple virulence determinants [19,188]. Disruption of QS therefore represents an anti-virulence strategy that attenuates pathogenicity without exerting direct bactericidal pressure, offering a potential means to limit antimicrobial resistance [189]. 

Several small-molecule QS inhibitors targeting the Agr system have demonstrated preclinical efficacy. Savirin selectively inhibits AgrA, suppressing RNAIII and virulence gene expression and reducing lesion formation in murine infection models, although its activity is strain dependent [189,190]. Staquorsin, a more stable Savirin analog, exhibits improved AgrA binding and effectively downregulates hemolysins, phenol-soluble modulins, and RNAIII in both MSSA and MRSA, without affecting bacterial viability [191]. Ambuic acid, a fungal metabolite, inhibits AgrC, thereby blocking AIP signaling and virulence expression. Notably, it selectively targets *S. aureus* while preserving commensal staphylococci and has been shown to reduce inflammation and bacterial burden *in vivo* [192]. 

In addition to QS-targeted approaches, bacteriophage-based therapies have emerged as a promising strategy for difficult-to-treat bone infections. Bacteriophages directly infect bacteria and can be classified as lytic or lysogenic, with lytic phages being preferred therapeutically due to their rapid bacterial killing and lack of genomic integration [193,194]. Phages possess several advantages, including specificity, the ability to penetrate infected tissues, activity against intracellular bacteria, and synergistic effects with antibiotics. In experimental models of MRSA-induced osteomyelitis, phage cocktails significantly improved clinical, histological, and radiological outcomes, including reduced inflammation and enhanced new bone formation [195]. Phage-derived lysins, such as PlySs2, have also demonstrated antibacterial activity and synergy with vancomycin, reducing bacterial loads in murine models of prosthetic joint infection [196]. Combined phage–antibiotic therapy has been shown to eradicate implant-associated infections caused by MRSA and *Pseudomonas aeruginosa* in animal models, whereas monotherapy was ineffective [197]. 

Systematic reviews have emphasized the ability of phages to degrade biofilms in infections beyond osteomyelitis, largely due to lysins and depolymerases [198]. Clinical trials in prosthetic joint infections report significantly improved outcomes when phage therapy is combined with antibiotics, achieving high response rates with minimal adverse effects [199]. Consistently, multicenter retrospective analyses of refractory infections, including osteomyelitis, confirmed favorable clinical outcomes with adjunctive phage therapy [200].

In contrast to the many promising advantages of bacteriophages, several limitations remain. The therapeutic application of bacteriophages is limited by their restricted spectrum of activity, which may reduce efficacy in polymicrobial infections, a common clinical scenario. Lysogenic phages can also promote horizontal gene transfer, including genes coding for toxins and antibiotic resistance genes. In addition, phage therapy preparations are complex as they contain both proteins and nucleic acids, which complicates standardization, quality control, and reliable assessment of therapeutic efficacy [201]. Another drawback is the emergence of bacterial resistance to bacteriophages. The broader use of phage therapy is also limited by uncertain pharmacokinetics, rapid degradation, and challenges in dosing assessment. Additionally, this therapy may occasionally trigger immune reactions. Finally, despite promising preclinical results, the lack of robust clinical trial data further restricts their broad use in clinical practice [201].

Table 2 provides a summary of studies focusing on the use of bacteriophage therapy in the treatment of bone infections.

### 3.3. Non-Antibiotic Antimicrobials

#### 3.3.1. Silver-Based Compounds

Among non-antibiotic antimicrobials, silver-based compounds have been extensively explored. *In vitro* studies have shown the efficacy of silver ions and nanoparticles in infections related to osteomyelitis [202]. *In vivo*, silver-ion-doped calcium phosphate beads eradicated infection and promoted bone formation in a rabbit model of chronic osteomyelitis, outperforming calcium phosphate alone or vancomycin-loaded beads [203]. Similarly, silver nanoparticle (AgNP)-loaded bone cement reduced bacterial burden and inflammation in rat models of MRSA osteomyelitis, although therapeutic benefit was not consistently superior when AgNPs were added to standard surgical and antibiotic treatment [202,204]. Silver-coated implants have also reduced bacterial load and osteolysis in murine femoral infection models, even under anaerobic conditions [205]. Clinical evidence for silver remains limited but encouraging. In patients with refractory chronic osteomyelitis, adjunctive use of electrically activated silver dressings following surgical debridement resulted in stable wound healing and successful union in previously treatment-resistant cases [206]. Case reports further describe successful infection eradication and bone healing using silver-coated fixation devices after repeated treatment failure [207]. In immunocompromised populations, silver-coated tumor prostheses significantly reduced postoperative and chronic periprosthetic infection rates compared with uncoated implants, particularly in revision settings [208].

#### 3.3.2. Povidone-Iodine (PVP-I)

Povidone-iodine (PVP-I) represents another well-established non-antibiotic antimicrobial. Iodine rapidly penetrates microorganisms and induces oxidative damage to proteins, nucleotides, and lipids, conferring broad-spectrum activity against pathogens commonly involved in bone infections, including *S. aureus* and *P. aeruginosa* [209]. Its efficacy has been demonstrated in multiple *in vitro* studies [210,211,212,213]. *In vivo*, iodine-coated titanium implants retained antimicrobial activity for up to eight weeks, reducing bacterial colonization and preventing infection without impairing osseointegration [214]. Consistent clinical data show that iodine-coated implants are effective for both prevention and treatment of orthopedic infections, with high cure rates, preserved thyroid function, excellent implant integration, and minimal adverse events across several prospective and retrospective studies [215,216,217]. 

#### 3.3.3. Antimicrobial Peptides (AMPs) 

Antimicrobial peptides (AMPs) are endogenous defense molecules with antimicrobial, immunomodulatory, and tissue-protective functions [218]. The function of AMPs is closely linked to their structure. Specifically, they are generally classified as linear, helical, β-structured, mixed α/β, or cyclic/complex peptides [219]. 

AMPs represent a promising solution in the battle against infectious diseases, as they exhibit antibacterial activity against both Gram-negative and Gram-positive bacteria, as well as, antiviral, antifungal, and antiparasitic activity, making them an active field of research, with over 5000 described [220]. Preclinical studies indicate that AMP-based implant coatings can prevent bacterial colonization without compromising bone healing. For example, the peptide RRP9W4N inhibited bacterial attachment on titanium implants *in vitro* and demonstrated safety for osseointegration in a rabbit tibial mode [221]. Polymer–AMP hybrid coatings incorporating chicken cathelicidin-2 (CATH-2) have similarly shown strong antibacterial efficacy without cytotoxicity *in vitro* [222]. However, human data for AMP-based strategies are currently lacking.

Although AMPs represent a promising strategy against antimicrobial resistance, several limitations remain unsolved. A major challenge is the poor translation of *in vitro* activity to *in vivo* efficacy, largely due to complex physiological mechanisms. In addition, routes of administration remain problematic because of low oral bioavailability, limited metabolic stability, and rapid systemic degradation. Finally, high production costs combined with insufficient clinical and toxicological data further distort the broader development and clinical application of AMPs [219,223].

#### 3.3.4. Bacteriocins

Bacteriocins are antimicrobial peptides produced by bacteria and inhibit the growth of strains of the same or closely related species [216]. A titanium implant coated with inactivated *Lactobacillus casei* biofilm achieved near-complete antibacterial efficacy against MRSA while simultaneously promoting osteogenesis through macrophage activation [224]. Lysostaphin-coated implants significantly reduced bacterial colonization and inhibited infection development in a minipig model, highlighting potential utility in implant-associated infections [225]. To date, these approaches remain unsupported by clinical trials.

#### 3.3.5. Synthetic Antimicrobial Polymers

Synthetic antimicrobial polymers represent another emerging strategy. Bioerodible polymer coatings combined with antibiotics such as levofloxacin or tigecycline have demonstrated sustained drug release and effective prevention of implant-associated infections in animal models [226,227]. Other polymeric coatings, including polyvinyl alcohol (PVA) and multilayer montmorillonite-based systems, reduced bacterial adhesion, limited inflammation, and improved bone healing *in vivo* without cytotoxicity [228,229]. It is important to highlight that, the antimicrobial function of synthetic antimicrobial polymers is strongly influenced by polymer properties, including charge density, molecular weight, hydrophobicity, and architecture, which pose significant challenges to their broader use [230]. Additional limitations include potential cytotoxicity and environmental concerns related to non-biodegradability. These factors must be carefully assessed to better evaluate the suitability of antimicrobial polymers for clinical application [230].

#### 3.3.6. Honey and Essential Oils

Honey constitutes another promising adjunctive therapy for osteomyelitis, Its anti-inflammatory, antioxidant, and antimicrobial properties may support bone healing by reducing inflammation, promoting angiogenesis, and limiting bacterial colonization [231]. Animal studies suggest that honey, particularly when incorporated into biomaterial scaffolds, can reduce infection and support bone regeneration; however, efficacy varies with formulation and delivery method, and human data remain scarce [232].

Finally, essential oils have demonstrated antimicrobial activity against orthopedic pathogens *in vitro*. Compounds derived from tea tree, geranium, rosemary, eucalyptus, and lavender oils inhibit *S. aureus* and *S. epidermidis*, likely through bioactive constituents such as terpinen-4-ol, citronellol, and geraniol [233,234]. Nevertheless, the absence of *in vivo* and clinical studies currently limits their translational relevance.

Table 3 provides an overview of the antimicrobial approaches for bone infections, emphasizing their biological mechanisms, clinical applicability, and translational readiness.

## 4. Biomarkers and Personalized Systems-Based Approaches

Assessment of treatment failure in osteomyelitis relies primarily on clinical history and physical examination, supported by imaging and selected inflammatory biomarkers. Commonly evaluated markers include erythrocyte sedimentation rate (ESR), C-reactive protein (CRP), procalcitonin (PCT), pro-inflammatory cytokines (IL-6, IL-8, TNF-α), and chemokines such as MCP-1 and MIP-1α. These markers are typically measured at diagnosis and after therapy completion to establish a new baseline; routine weekly monitoring during antibiotic treatment is generally unnecessary. Although several studies associate trends in ESR and CRP with treatment response [235,236,237,238], others show no correlation [239], and even when associations exist, these markers rarely provide additional value beyond clinical assessment [240]. ESR often declines slowly and may remain elevated for weeks to months, limiting its utility for short-term monitoring [237]. 

PCT and IL-6 may aid diagnosis and treatment monitoring, as reductions in CRP, ESR, PCT, and IL-6 have been observed during effective therapy [238]. However, PCT appears more useful for ruling out infection than confirming osteomyelitis, and its diagnostic sensitivity remains limited despite improved performance at lower cut-off values [241,242,243]. Biomarker performance is strongly influenced by host factors. In diabetes, ESR and CRP demonstrate higher predictive value, while ESR sensitivity and specificity are reduced in patients without peripheral neuropathy; CRP performance remains relatively stable regardless of neuropathy status [244]. These findings support individualized biomarker interpretation based on host characteristics rather than universal thresholds [245].

Additional biomarkers may improve diagnostic and prognostic accuracy. Elevated MCP-1 correlates with inflammatory activity and recurrence risk in osteomyelitis [246,247], while hypocalcemia independently predicts mortality in severe acute cases requiring intensive care [248]. Multi-biomarker panels, combining inflammatory, infectious, and osteoimmunological markers (e.g., CTx-II, TGF-α, MCP-1, sCD14-ST, SuPAR, CCL2, IL-6, and RANKL/OPG ratio), enhance discrimination between osteomyelitis and septic arthritis and improve treatment monitoring [246,249,250]. Early normalization of SuPAR and CCL2 supports their prognostic value, whereas prolonged IL-6 elevation limits early response assessment [245]. Oxidative stress markers, including reactive oxygen species, malondialdehyde, and protein carbonyls, reflect disease severity and contribute to bone loss by inhibiting osteoblasts and activating osteoclasts [251,252,253,254,255,256]. Reduced paraoxonase-1 further indicates impaired antioxidant defense and increased infection susceptibility [253,254]. In diabetic foot osteomyelitis, combining nCD64 (high sensitivity) with ESR (high specificity) improves early diagnostic accuracy [257].

Advances in molecular diagnostics have transformed host–pathogen profiling in osteomyelitis. Culture-independent techniques reveal complex microbial communities and host responses within bone and periprosthetic environments, enabling classification into microbial “types” associated with distinct inflammatory signatures [258]. Intracellular *S. aureus* induces host transcriptional remodeling by downregulating epigenetic repressors such as NuRD and PRC1 complexes, promoting persistence and inflammation [259]. Metatranscriptomics (MT) and metagenomic next-generation sequencing (mNGS) outperform conventional cultures by detecting metabolically active pathogens and resistance genes, with markedly higher sensitivity across osteomyelitis subtypes, especially in culture-negative or antibiotic-exposed cases [260,261,262,263,264].

Host transcriptomic profiling further distinguishes infectious osteomyelitis from autoinflammatory bone disorders such as CRMO and CNO, where inflammation occurs without pathogens [265]. Endotyping approaches differentiate bone-restricted from systemic phenotypes and highlight the role of host susceptibility, including gut microbiome dysbiosis, in amplifying bone inflammation through barrier disruption and systemic cytokine activation [266]. Recognition of host-driven osteomyelitis variants underscores the need to distinguish immune-mediated disease from infection to guide immunomodulatory rather than antimicrobial therapy [267,268]. Thus, a modern framework for diagnosis and treatment requires a combined host–pathogen profile that includes microbial detection, transcriptional activity, and host immune-epigenetic responses within the clinical context [261].

Artificial intelligence and machine-learning approaches are increasingly applied to osteomyelitis diagnostics and management. Bioinformatic analyses have identified diagnostic biomarkers and molecular subtypes in CNO, supporting precision classification and targeted therapy development [269]. Deep-learning models integrating laboratory data can predict culture results, optimize treatment timing, and monitor relapse risk, particularly in pediatric osteomyelitis [270]. AI-assisted analysis of CT and MRI improves detection of chronic osteomyelitis and informs surgical decision-making, potentially reducing recurrence rates [271,272]. Similarly, neural network models incorporating dynamic CRP trends outperform static baseline predictors in forecasting recurrence of pyogenic vertebral osteomyelitis [273].

Despite these advances, ethical, legal, and practical challenges remain. AI implementation requires robust data security, anonymization, regulatory compliance, and transparent oversight to ensure patient safety [274]. Highly sensitive molecular diagnostics risk identifying clinically irrelevant organisms, potentially leading to overtreatment, while high costs and reliance on unvalidated biomarkers raise concerns regarding consent, equity, and clinical applicability. Overcoming these challenges will require multidisciplinary collaboration to responsibly integrate precision diagnostics and AI-driven tools into routine osteomyelitis care [275]. 

## 5. Challenges, Controversies and Future Directions

Although alternative therapies have shown promise in treating bone infections, the development of novel therapies is hindered by multiple challenges, including the mismatch between preclinical and clinical findings, as well as funding and regulatory barriers. Preclinical models often fail to represent the complex dynamics of human bone infections. Simplified *in vitro* systems inadequately predict antimicrobial performance in clinical settings [276]. While small animal models are cost effective and easy to handle, anatomical and physiological differences reduce their translational relevance. Conversely, large animal models replicate more accurately the characteristics of human bone but are more expensive and are associated with strict ethical considerations [277]. 

Inconsistent translation is also driven by agent-specific limitations. Many antimicrobial peptides with strong *in vitro* activity have failed in clinical development due to reduced efficacy in clinically relevant environments or lack of superiority over standard antibiotics [278,279]. A similar pattern is evident in the development of antibacterial coatings for orthopedic implants. Despite the promising experimental performance of several coating technologies, only a limited number have reached clinical use. Many materials were unsuitable due to cytotoxicity, immunoreactivity or interference with bone healing. However, even the agents that meet the safety requirements are often prevented from large-scale clinical trials due to financial or regulatory barriers [280]. Current requirements for market approval of such agents are challenging and manufacturers must provide clinical data on safety, performance and a favorable benefit–risk profile [281]. Comparable regulatory and translational challenges are evident for other non-antibiotic therapies, including bacteriophage-based approaches [282]. An additional challenge in clinical evaluation is the lack of standardized endpoints. Although infection eradication, recurrence or persistence are commonly reported definitions and follow-up periods vary widely, and functional outcomes or patient-reported measures such as quality of life are rarely assessed. This heterogeneity complicates cross-study comparisons and may contribute to inconsistent interpretation of treatment success or failure.

Current clinical guidelines continue to prioritize systemic antibiotic therapy in combination with surgical management for PJIs or FRIs [283,284]. Adjunctive local antimicrobial strategies are acknowledged within these guidelines but are supported only by limited or conditional recommendations [283,285]. For example, guidelines from the American Academy of Orthopaedic Surgeons (AAOS) note that the routine use of antibiotic-loaded bone cement has not been shown to significantly reduce the risk of PJI in patients undergoing cemented total knee arthroplasty. Nevertheless, the inclusion of local antimicrobial approaches within current guidelines suggests a recognized potential role, which may be more clearly defined and expanded as higher-quality clinical evidence becomes available [283]. Non-systemic approaches, such as local antibiotic therapy, may be particularly beneficial in the management of chronic and recurrent BJIs, as available clinical evidence indicates that their use is not associated with an increased emergence of antimicrobial resistance [286], while surgical treatment in these settings is often complex and accompanied by a high risk of complications [287,288]. In a similar way, certain individuals with a high suspicion of biofilm-associated infection, particularly in implant-related settings dominated by staphylococcal pathogens, alternative approaches such as anti-biofilm biomaterials and bacteriophage-based therapies may be especially relevant, as these strategies are designed to disrupt established biofilms, limit bacterial protection from host defenses and antibiotics, and reduce reliance on diffusible antimicrobial agents that may promote resistance [289,290]. 

Clinicians should therefore be attentive to patients at high risk of recurrent osteomyelitis, those with an increased likelihood of treatment-related complications such as hepatotoxicity or nephrotoxicity, and individuals with infections involving resistant mechanisms, and should consider the hypothesis of early incorporation of alternative or adjunctive non-systemic antimicrobial strategies within the overall treatment plan.

## 6. Conclusions

Systemic antibiotics alone often fail to eradicate bone infections because of limited bone vascularization, bacterial persistence, and increasing antimicrobial resistance. These challenges have driven the development of innovative alternative strategies, including local antimicrobial delivery, immunomodulation, bacteriophage therapy, and other non-antibiotic approaches. Antimicrobial peptides provide rapid and broad antimicrobial activity and exhibit a low tendency to induce resistance; however, their clinical advancement is restricted by limited durability in biological environments and challenges associated with formulation and large-scale production. Synthetic polymer-based systems enable localized and sustained antimicrobial delivery and offer design flexibility to tailor release profiles and biological interactions, although their long-term *in vivo* behavior and material-dependent safety profiles require further clarification. Bacteriophage-based approaches offer high pathogen specificity and effective biofilm disruption, yet their therapeutic applicability is influenced by host range restrictions, immune interactions, and the need for individualized matching to bacterial strains. Nanoparticle-based strategies, particularly those relying on biodegradable polymeric or lipid platforms, combine targeted delivery with controlled release and reduced off-target exposure, but their performance remains closely linked to formulation parameters and manufacturing consistency.

Future advances will rely on refining material design, improving reproducibility across production batches, and aligning preclinical evaluation with clinically relevant outcomes to support rational, context-specific application.

## Figures and Tables

**Table 1:** Local Delivery Systems.

Local Delivery System	Advantages	Disadvantages
PMMA (Polymethyl methacrylate)	- Good biocompatibility - Can sustain high local antibiotic concentrations - Proven effectiveness in chronic osteomyelitis - Allows for combination of antibiotics - Reduces infection recurrence	- Non-biodegradable; requires surgical removal - Limited antibiotic options (heat-stable, powder form) - Variable elution time - Reduced mechanical strength with high antibiotic load - Risk of bacterial resistance - Spacer-related mechanical complications
Calcium Sulfate (CaS)	- Biocompatible and biodegradable - Sustained antibiotic release - Achieves high local concentrations without systemic toxicity - Promotes bone regeneration	- Low mechanical strength - Risk of hypercalcemia - Postoperative wound drainage - Limited efficacy in immunocompromised patients - Variable degradation rates
Hydroxyapatite (HA)	- Excellent biocompatibility and bioactivity - Promotes bone regeneration - Effective carrier for water-soluble antibiotics	- Crystallizes and coagulates rapidly *in vivo* when used alone - Requires combination with other materials for stability - Potential wound complications and infection persistence - Occasional need for revision surgeries
Hydrogels	- High biocompatibility and biodegradability - Mimic extracellular matrix - Support bone tissue formation - Enable targeted and sustained drug release - Reduce systemic toxicity and bacterial resistance - Proven safety as implant coatings - Useful for both prevention and treatment of infection	- Limited robust clinical evidence -May require optimization for mechanical stability - Effectiveness varies depending on formulation and infection type
Antibiotic-Impregnated Bone Grafts (AIBGs)	- Combine structural support with infection control - Support bone regeneration and incorporation - Allow broad antibiotic selection - Long-term infection-free outcomes - Effective in trauma and revision surgeries	- Variable antibiotic elution when combining drugs - Preparation-dependent release kinetics - Potential risk of reinfection in impaction grafts - Limited availability of standardized protocols
Nanoparticles	- Nanoscale size allows precise, controlled drug release - Promote bone regeneration and tissue integration - Biodegradable—no removal surgery required - Reduce systemic toxicity - Effective against biofilm and resistant bacteria	- Possible complications (e.g., refracture, infection recurrence, wound necrosis) - Manufacturing complexity - Long-term safety data limited - Cost and scalability challenges

PMMA: Poly(methyl methacrylate); HA: Hydroxyapatite; AIBGs: Antibiotic-Impregnated Bone Grafts; CaS: Calcium Sulfate.

**Table 2:** Bacteriophage-treatment studies.

Name of the Study	Model/Subjects	Infection Type	Internation/Comparison	Main Outcomes
Kishor et al., 2016 [195]	*In vivo*—22 rabbits	MRSA osteomyelitis	Local administration of a 7-phage cocktail	Significant clinical, histopathological and radiological improvement and new bone formation. Safe and effective
Sosa et al., 2020 [196]	*In vivo* murine models and *in vitro* assays	Prosthetic joint osteomyelitis caused by *S. aureus*	PlySs2 lysin—alone or combined with vancomycin	Synergistic action with vancomycin—reduced bacteria load in periprosthetic tissue and on implant surfaces
Mobarezi et al., 2025 [198]	Systematic review	Various infections	Phages, Lysins and Depolymerases	Highlighted biofilm degradation ability of phages—mostly *in vitro* data—more animal/human research required
Yilmaz et al., 2013 [197]	*In vivo* and rat models	Implant-related infection (MRSA & *P. aeruginosa*)	4 subgroups: control, phage only, antibiotic only, phage + antibiotic	In MRSA group: biofilm eradicated only in combined phage + antibiotic group. In *P. aeruginosa*: reduced CFU in all treatments, but biofilm thickness unchanged.
Fedorov et al., 2023 [199]	Clinical trial—Human patients	PJI	Bacteriophage therapy + antibiotics vs. antibiotics alone	Combined therapy improved outcomes (95.5% response); faster drop in inflammation markers; mild, transient adverse events (fever).
Pirnay et al., 2024 [200]	Multicenter, retrospective study—humans	Difficult-to-treat infections (including bone infections)	Bacteriophage therapy + antibiotics	Favorable clinical outcomes; supports combined approach in complex infections.

CFU: Colony Forming Unit; IL: Interleukin; MRSA: Methicillin-Resistant *Staphylococcus aureus*; *P. aeruginosa*: *Pseudomonas aeruginosa*; PJI: Prosthetic Joint Infection; *S. aureus*: *Staphylococcus aureus*.

**Table 3:** Antimicrobial strategies.

Strategy	Main Mechanism(s) of Action	Target Pathogens/Context	Key Advantages	Main Limitations	Technological/Clinical Maturity
Immunomodulatory approaches (MSCs, interleukins and macrophages)	Regulation of innate and adaptive immunity; suppression of excessive inflammation; promotion of osteogenesis and angiogenesis	Chronic osteomyelitis; impaired healing; inflammatory dysregulation	Supports bone regeneration; modulates host response	Variability in cell source and donor; limited homing efficiency; lack of standardized protocols; regulatory and ethical constraints	Preclinical to early clinical (fracture healing, nonunion); limited infection-specific trials
Vaccination strategies	Induction of pathogen-specific humoral and cellular immunity	*S. aureus* (including MRSA); infection prevention	Potential reduction in infection burden and antibiotic reliance	Failure to demonstrate clinical protection; inconsistent efficacy; possible new colonization	Preclinical to phase II/III trials; no approved vaccines
Quorum sensing/anti-virulence therapies	Inhibition of bacterial communication and virulence gene expression without bactericidal pressure	Biofilm-associated *S. aureus* infections	Reduces pathogenicity without strong selective pressure; preserves commensals	Mostly preclinical; strain-dependent effects; limited *in vivo* data	Preclinical
Bacteriophages and phage-derived lysins	Targeted bacterial lysis; biofilm degradation via lysins and depolymerases	Refractory, biofilm-dominated infections; MRSA; *P. aeruginosa*; PJI and osteomyelitis	High specificity; biofilm penetration; synergy with antibiotics	Narrow host range; resistance development; immune reactions; standardization and dosing challenges	Preclinical to early clinical
Silver-based compounds	Broad-spectrum antimicrobial activity; disruption of bacterial metabolism and biofilms	Chronic and refractory infections; implant-associated infections; MRSA	Activity against resistant bacteria; coating and local delivery options; tested in immunocompromised individuals	Lack of superiority when combined with standard care; limited clinical data	Preclinical to limited clinical use
Povidone-iodine (PVP-I)	Oxidative damage to proteins, lipids, and nucleic acids	Prevention and treatment of implant-related infections; *S. aureus* and *P. aeruginosa*	Broad-spectrum activity; preserved osseointegration; favorable clinical safety	Mainly implant/coating-based	Advanced preclinical and clinical use
AMPs	Membrane disruption; immunomodulation	Gram-positive and Gram-negative bacteria, viruses, parasitic infections	Broad activity	Poor *in vivo* stability; limited bioavailability; high production costs; lack of clinical data	Preclinical
Bacteriocins	Narrow-spectrum antibacterial activity against related strains	Implant-associated *S. aureus* infections	High specificity; reduced impact on microbiota	Lack of clinical trials; narrow activity spectrum	Preclinical
Synthetic antimicrobial polymers	Contact-killing or drug release depending on polymer properties	Implant-associated and local infections	Tunable properties; sustained release; reduced adhesion	Cytotoxicity risk; environmental concerns; structure-dependent performance	Preclinical
Honey and essential oils	Antimicrobial, anti-inflammatory, and antioxidant effects	Adjunctive therapy; experimental settings *S. aureus* and *S. epidermidis*	Multimodal biological activity	Variable efficacy; limited *in vivo* and clinical data	Preclinical

Abbreviations: AMPs, antimicrobial peptides; BJI, bone and joint infections; CFU, colony-forming unit; FRI, fracture-related infection; IL, interleukin; MRSA, methicillin-resistant *Staphylococcus aureus*; MSCs, mesenchymal stem cells; MSSA, methicillin-susceptible *Staphylococcus aureus*; PJI, prosthetic joint infection; PLGA, poly(lactic-co-glycolic acid); PVA, polyvinyl alcohol; QS, quorum sensing; PVP-I, povidone-iodine.

## Data Availability

No new data were created or analyzed in this study.

## Supplementary Materials

The following supporting information can be downloaded at: https://www.mdpi.com/article/10.3390/pathogens15020201/s1, File S1: PRISMA 2020 flow diagram and PRISMA Checklist.

## Abbreviations

AIBGs	Antibiotic-Impregnated Bone Grafts
AgNP	Silver Nanoparticles
AI	Artificial Intelligence
ALBC	Antibiotic-Loaded Bone Cement
AMP	Antimicrobial Peptides
AAOS	American Academy of Orthopaedic Surgeons
BJIs	Bone and Joint Infections
CaS	Calcium Sulfate
CFU	Colony-Forming Unit
CNO	Chronic Nonbacterial Osteomyelitis
CRMO	Chronic Recurrent Multifocal Osteomyelitis
CRP	C-Reactive Protein
CT	Computed Tomography
DFI	Diabetic Foot Infection
ECM	Extracellular Matrix
*E. coli*	*Escherichia coli*
EGFR	Epidermal Growth Factor Receptor
EPS	Extracellular Polymeric Substances
ESR	Erythrocyte Sedimentation Rate
FRI	Fracture-Related Infection
HA	Hydroxyapatite
HBOT	Hyperbaric Oxygen Therapy
ICM	International Consensus Meeting
IL	Interleukin
MCP-1	Monocyte Chemoattractant Protein-1
MRI	Magnetic Resonance Imaging
MRSA	Methicillin-Resistant *Staphylococcus aureus*
MSCs	Mesenchymal Stem Cells
MT	Metatranscriptomics
NK	Natural Killer (cells)
OPG	Osteoprotegerin
PCT	Procalcitonin
PJI	Prosthetic Joint Infection
PLGA	Poly(lactic-co-glycolic acid)
PMMA	Polymethyl Methacrylate
PMNs	Polymorphonuclear Neutrophils
PRISMA	Preferred Reporting Items for Systematic Reviews and Meta-Analyses
PVA	Polyvinyl Alcohol
PVP-I	Povidone-Iodine
QS	Quorum Sensing
RANK-L	Receptor Activator of Nuclear Factor κB Ligand
ROS	Reactive Oxygen Species
*S. aureus*	*Staphylococcus aureus*
SCVs	Small-Colony Variants
SuPAR	Soluble Urokinase Plasminogen Activator Receptor
TNF-α	Tumor Necrosis Factor Alpha
TLR4	Toll-Like Receptor 4
VEGF	Vascular Endothelial Growth Factor
VRE	Vancomycin-Resistant Enterococci
