# Supplementary material for: Managing Bone Infections Beyond Systemic Antibiotics: A Scoping Review
Source: Pathogens. 2026 Feb 11;15(2):201. doi: 10.3390/pathogens15020201 (PMC12943792; doi:10.3390/pathogens15020201)
Supplement: Supplementary file 1 [file pathogens-15-00201-s001.zip › Supplementary File S1/PRISMA 2020 flow diagram.pdf]

METHODOLOGY

A literature search was conducted by five independent reviewers in PubMed using Medical Subject Headings (MeSH) and free-text terms related to bone infections, their pathophysiology, and emerging therapeutic and diagnostic strategies. The MeSH terms included *Osteomyelitis; Bone Diseases, Infectious diseases and and Bones; Prosthesis-Related Infections; Fracture-Related Infections; Surgical Wound Infection; Biofilms; Bacterial Adhesion; Bacterial Persistence; Drug Resistance, Bacterial; Anti-Bacterial Agents; Administration, Local; Drug Delivery Systems; Biocompatible Materials; Polymethyl Methacrylate; Calcium Sulfate; Hydroxyapatites; Hydrogels; Bone Transplantation; Nanoparticles; Immunotherapy; Immunomodulation; Mesenchymal Stem Cells; Cytokines; Interleukins; Macrophages; Bacteriophages; Quorum Sensing; Virulence Factors; Antimicrobial Peptides; Silver; Iodine Compounds; Biomarkers; Inflammation Mediators; Molecular Diagnostic Techniques; High-Throughput Nucleotide Sequencing; Transcriptome; Machine Learning;* and *Artificial Intelligence*. These terms were combined with relevant keywords using Boolean operators (AND/OR).

The initial search identified 1,237 records. After removal of 45 duplicate records and 524 records marked as ineligible by automation tools**,** 668 records remained for title and abstract screening. During this stage, 201 records were excluded based on irrelevance to bone infections or failure to meet predefined inclusion criteria.

A total of 467 full-text articles were assessed for eligibility. Of these, 200 reports were excluded for the following reasons: absence of bone involvement (n = 57), exclusive focus on soft-tissue infections (n = 39), insufficient methodological detail or outcome data (n = 49), or other predefined exclusion criteria (n = 32). Ultimately, 290 studies and reports met the inclusion criteria and were included in the qualitative synthesis.

Study selection, screening, and inclusion were conducted in accordance with the PRISMA 2020 framework, as illustrated in Supplementary Figure 1. All screening stages were performed independently by the reviewers, with discrepancies resolved through discussion and consensus. Only studies published in English and involving human subjects or clinically relevant experimental models were considered eligible. Given the heterogeneity of study designs, interventions, and reported outcomes, a meta-analysis was not performed, and findings were synthesized narratively.

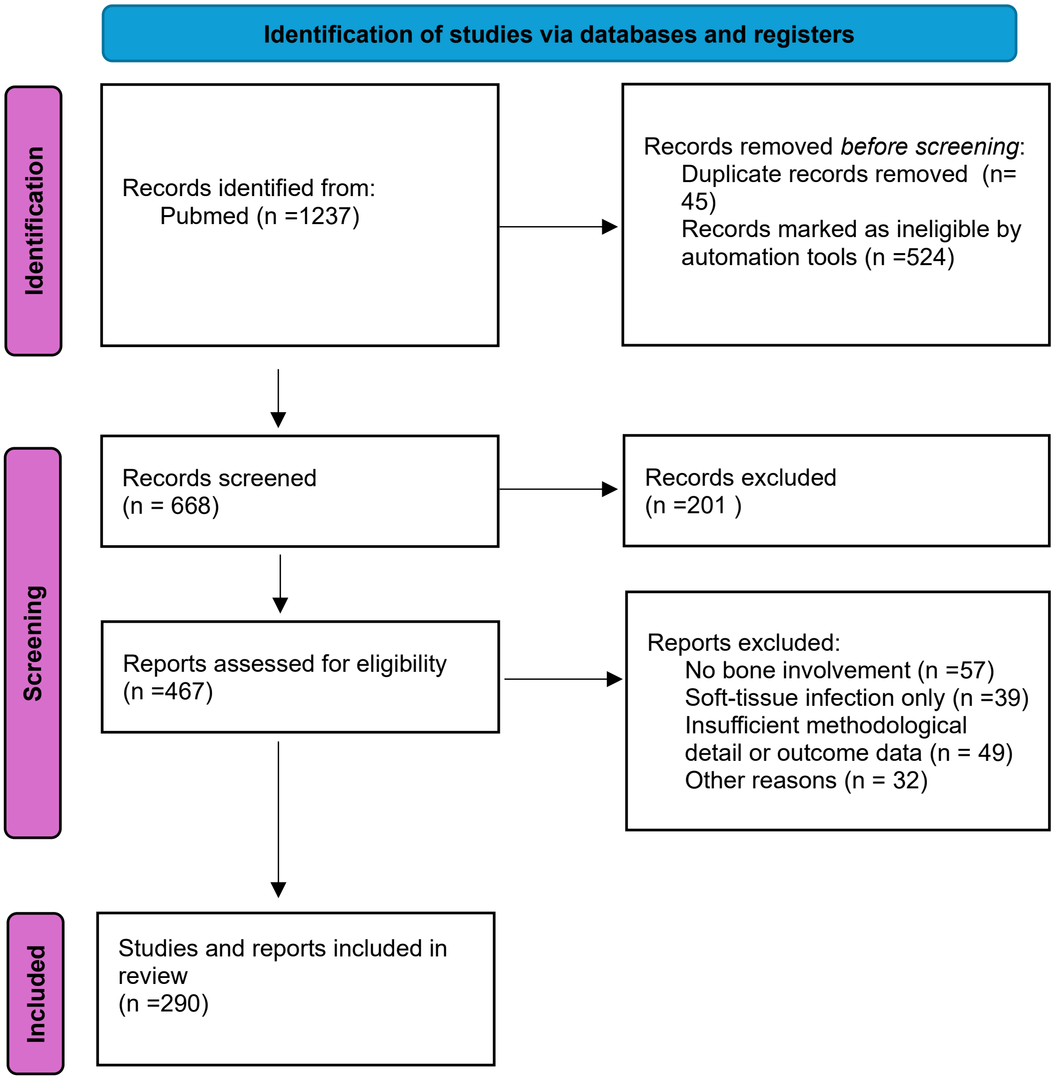

PRISMA 2020 flow diagram
